# In-Flight Tuning of Au–Sn Nanoparticle Properties

**DOI:** 10.1021/acs.langmuir.4c01656

**Published:** 2024-07-26

**Authors:** Pau Ternero, Calle Preger, Axel Christian Eriksson, Jenny Rissler, Julia-Maria Hübner, Maria E. Messing

**Affiliations:** † Department of Physics and NanoLund, Lund University, 221 00 Lund, Sweden; ‡ Department of Design Sciences and NanoLund, Lund University, 221 00 Lund, Sweden; § MAX IV Laboratory, Lund University, 221 00 Lund, Sweden; ∥ Faculty of Chemistry and Food Chemistry, TUD Dresden University of Technology, 01062 Dresden, Germany

## Abstract

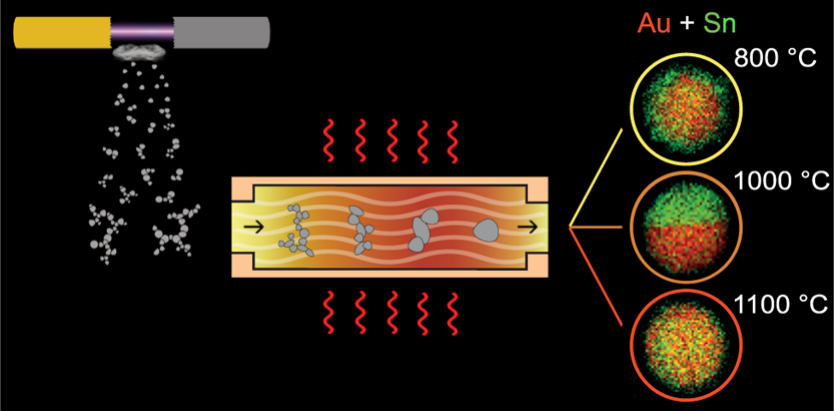

Multimetallic nanoparticles possess a variety of beneficial properties with potential relevance for various applications. These metallic nanoparticles can consist of randomly ordered alloys, which retain the properties of the constituting elements, or ordered intermetallics, which possess extended properties. Depending on the desired application, specific alloys or intermetallic compounds are required. However, it remains challenging to achieve particular morphologies, crystal structures, chemical compositions, and particle sizes because of the inherent complexity of nanoparticle synthesis. In this work, Au–Sn nanoparticles were synthesized using a continuous one-step gas-phase synthesis method that offers the possibility to anneal the nanoparticles in flight directly after generation to tune their properties. The bimetallic model system Au–Sn, comprising both alloys and intermetallic compounds, was studied in the temperature range of 300 to 1100 °C. The bimetallic Au/Sn ratio in the nanoparticles can be adjusted with in-flight annealing between 70/30 and 40/60 atomic %. While Au-rich alloys are obtained at lower temperatures, the increase in the annealing temperature leads to the formation of more Sn-rich intermetallic phases. Surface and size effects greatly influence particle morphologies and phase fractions. This research opens new opportunities for the synthesis of customized nanoparticles by temperature adjustment and particle size selection.

## Introduction

In the vast landscape of nanoscience, metallic nanoparticles attract significant attention due to their remarkable properties,^1,2^ which are distinct from their bulk counterparts by the additional influence of size, surface energy, and quantum effects. These properties facilitate applications in various fields, including biomedicine,^3,4^ optics,^5^ electronics,^6,7^ energy,^8,9^ and catalysis.^10,11^ Moving from monometallic to bi- or multimetallic nanoparticles, a larger versatility in compositions and atomic arrangements can be attained, which facilitates the expansion of their properties and in turn applications.^12^ Multimetallic nanoparticles can consist of randomly ordered alloys or structurally ordered intermetallic compounds.^13^ While alloys retain the properties of individual components,^14^ intermetallics are typically more thermodynamically stable and can offer superior properties compared to the constituting metals.^15,16^

Synthetic approaches commonly used to produce multimetallic nanoparticles include hydrothermal, solvothermal, sol–gel, electrochemical, or electroless methods.^17^ However, the design of nanoparticles is challenging due to the complex formation mechanisms involved,^18,19^ and various side products are encountered using these solution-based methods.^20^ The initial synthesis can be followed by annealing at elevated temperatures to further modify the properties of the nanoparticles, i.e., morphology, crystal structure, chemical composition, and size.^21,22^ Nevertheless, aggregation can occur during annealing, leading to a reduction in active surface areas.^23^ In particular, the synthesis of tunable multimetallic nanoparticles has proven challenging in the past.^24^

Alternatively, spark ablation, as a gas-phase synthesis technique working at atmospheric pressure, is known for being simple, continuous, environmentally friendly, inexpensive, and solvent free, and is able to grant access to high-purity nanoparticles.^25^ This technique is particularly interesting due to its flexibility since it allows for tuning of nanoparticle properties by adjusting the synthesis parameters.^26−29^ Besides, the nanoparticles can be annealed in the gas phase directly after generation, avoiding the formation of aggregates.^30,31^ However, the influence of a rapid in-flight annealing on nanoparticle properties is material dependent and largely unknown.^32,33^ Hence, a better understanding of this process is required to facilitate a custom material synthesis. Furthermore, phase stability ranges, readily available in phase diagrams for bulk systems, remain challenging to obtain at the nanoscale.^34,35^

The bulk phase diagram of the bimetallic system Au–Sn^36^ comprises several alloy and intermetallic compounds over a wide compositional range, making this system suitable for evaluating the tunability of the in-flight annealing process at the nanoscale. Furthermore, Au–Sn nanoparticles are promising for several applications due to their electrocatalytic activity toward carbon dioxide reduction,^37^ catalytic and photocatalytic performance,^38,39^ tunable plasmonic properties at varying bimetallic composition,^40^ and hydrogen sensing capability.^41^

In this work, a continuous one-step gas-phase synthesis method based on spark ablation is applied. After generation, the nanoparticles are annealed in flight for about 5 s. Nanoparticle morphologies, crystal structures, and compositions are studied in the range of 300 to 1100 °C. The influence of the surface composition and particle size is investigated. The goal of the research is to enhance the understanding of nanoparticle formation mechanisms, including phase stability ranges, using in-flight annealing. This knowledge will allow for tailoring metallic nanoparticle properties for a broad range of applications.

## Experimental Section

**Figure 1:**
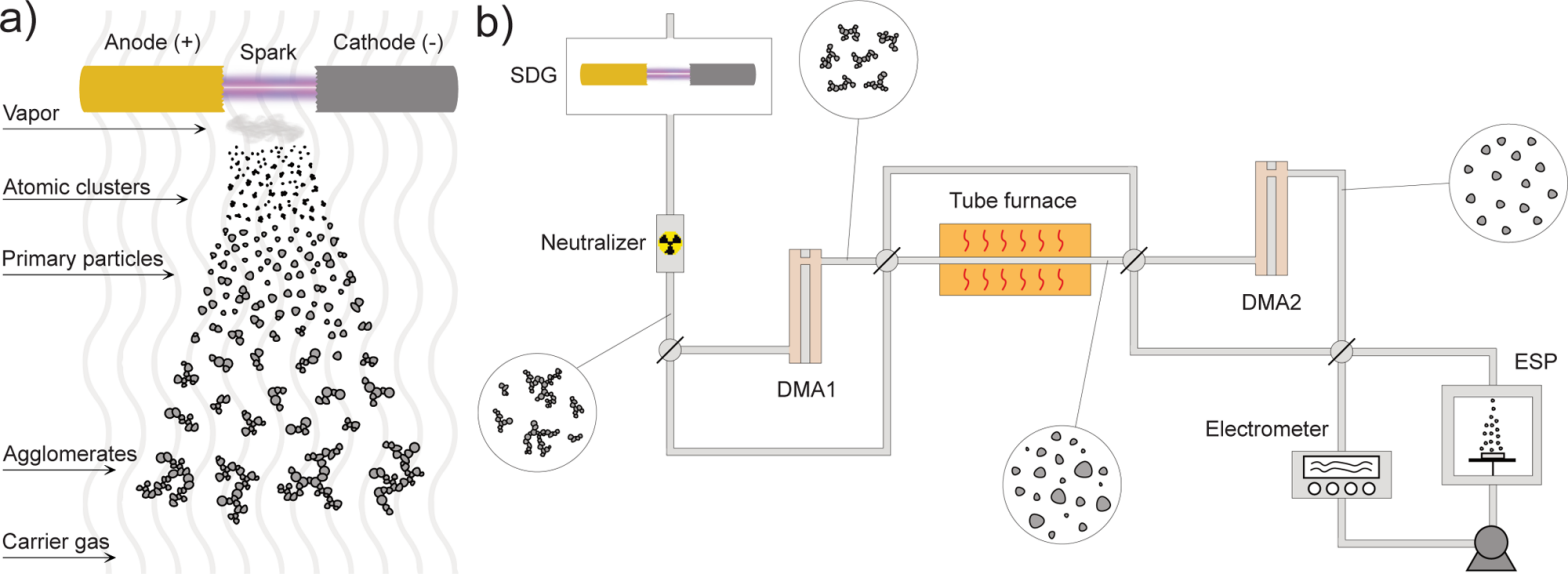
Schematic representation of (a) spark ablation synthesis and (b) the custom-build setup.

A spark discharge generator (SDG) was used to produce Au–Sn nanoparticles using atmospheric-pressure spark ablation synthesis^25^ (Figure 1a). A custom-built setup was employed for the subsequent annealing, size selection, and deposition of nanoparticles (Figure 1b). After generation, the in-flight annealing study was conducted in a tube furnace (Lenton LTF, ceramic tube Alsint 99.7 type C 799, length of 60 cm, and inner and outer diameters of 1.8 and 2.4 cm). When needed, the nanoparticles were size selected, based on their electrical mobility diameter, by means of a differential mobility analyzer (DMA). A detailed explanation of the process is found in the Supporting Information (SI).

The nanoparticles were analyzed right after production (online) or deposited on Si substrates using an electrostatic precipitator (ESP) for subsequent characterization (off-line). An electrometer (TSI 3086B) in combination with DMA2 was employed to record particle size distributions based on the electrical mobility diameter of the nanoparticles. In-flight X-ray photoelectron spectroscopy (XPS)^100^ was performed at the gas-phase endstation of the FinEstBeAMS beamline at MAX IV Laboratory to study the particle surface composition. Laser vaporizer aerosol mass spectrometry (AMS),^42^ measured in parallel with XPS, provided the Au/Sn ratio of the nanoparticles. The morphology and chemical composition of the nanoparticles were evaluated using scanning electron microscopy (SEM) (Zeiss GeminiSEM 500) with an energy-dispersive X-ray spectroscopy (EDS) detector (Oxford Instruments, Ultim Max, 170 mm^2^). The crystal structure of the samples was studied with powder X-ray diffraction (PXRD) in transmission mode (Stoe Stadi MP, Mythen 1k detector, Cu Kα radiation, λ = 1.54178 Å). Rietveld refinements of PXRD data were performed with the Jana2020 software^43^ to obtain the weight fractions of each phase for bulk samples. These phases were correlated with microscopic observations of individual agglomerates/nanoparticles using transmission electron microscopy (TEM) (Jeol JEM-3000F) with an EDS detector (Oxford Instruments, X-Max, 80 mm^2^). For each sample, about 20 distinct agglomerates/nanoparticles were analyzed at different locations of the TEM grid. The particular settings and procedures for each technique are available in the SI.

## Results and Discussion

### Nanoparticle Morphology

**Figure 2:**
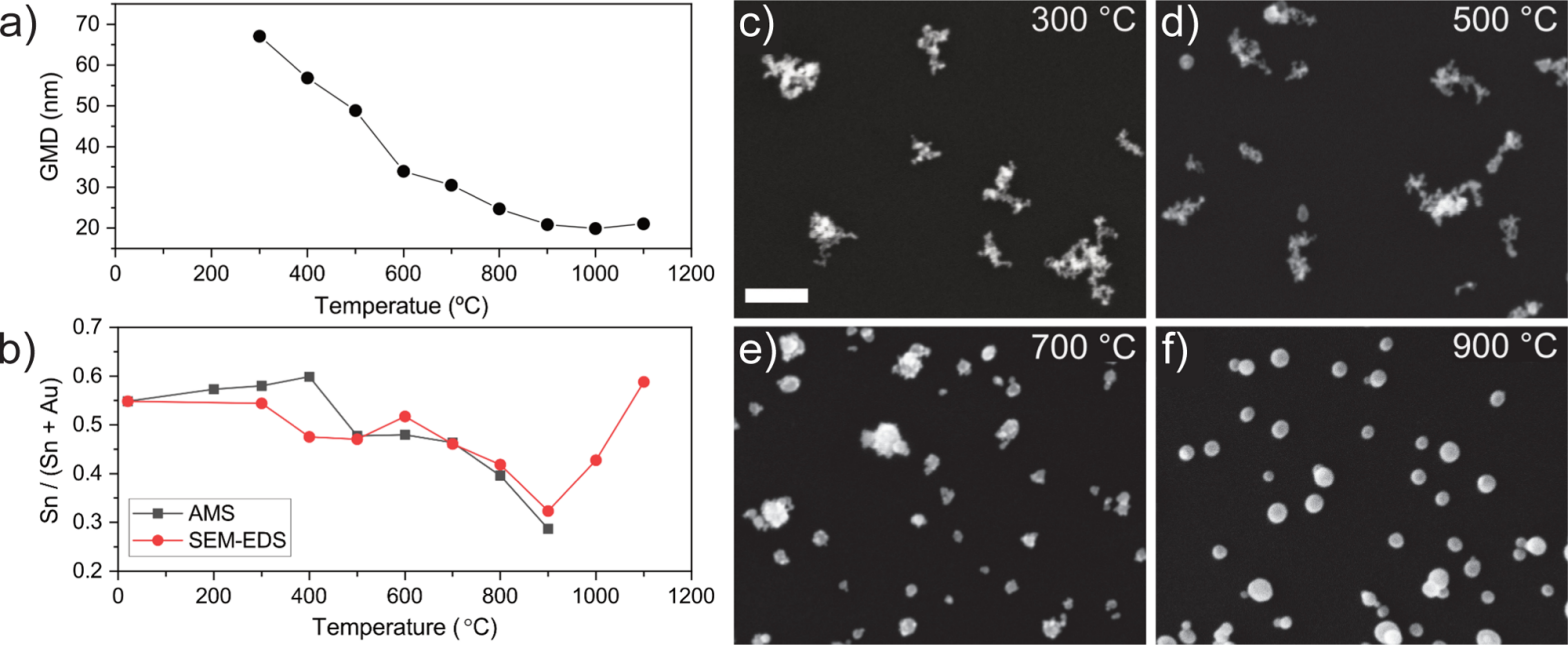
(a) GMD and (b) relative atomic composition of Sn as a function of the annealing temperature for Au–Sn nanoparticles measured with SEM-EDS and AMS. AMS-derived composition has been rescaled to match SEM-EDS at 20 °C, see the SI for details. SEM micrographs of the nanoparticles at (c) 300, (d) 500, (e) 700, and (f) 900 °C. Scale bar = 100 nm.

As-produced aerosol nanoparticles in spark ablation synthesis consist of fractal-like agglomerates with primary particle sizes of <10 nm.^44^ The in-flight annealing step after generation leads to sintering of agglomerates into spherical particles, reducing their overall mobility diameter.^30^ The geometric mean electrical mobility diameter (GMD) as a function of the annealing temperature (Figure 2a) was extracted from the measured size distributions (see SI, Figure S1 and Table S1). The morphology of the nanoparticles was investigated by SEM (Figure 2c–f).

From 300 to 600 °C, the mobility diameter of the nanoparticles decreases drastically as the temperature increases. Then, a slower change in particle size takes place up to 900 °C, where the sintering is completed since the particle size distributions at higher temperatures remain nearly constant. SEM micrographs agree with the general trend. The greatest change in particle sizes is observed in the range from 300 to 700 °C (Figures 2c–e). At 900 °C (Figure 2f), the particles are fully sintered, i.e., no further decrease in particle diameter takes place.

### Nanoparticle Bimetallic Composition

The relative atomic composition of Sn in Au–Sn nanoparticles was evaluated by AMS and SEM-EDS as a function of the annealing temperature (Figure 2b).

Au–Sn nanoparticles at room temperature correspond to the agglomerates as produced in the spark. The increase in temperature leads to a decrease in the relative amount of Sn up to 900 °C. The decrease in Sn content is attributed to the evaporation and condensation of Sn species in a temperature sink at the end of the tube furnace. Interestingly, after further increase in temperature, the relative amount of Sn starts to increase, and at 1100 °C, it is comparable to the starting value. This observation suggests the formation of more Sn-rich compounds at higher temperatures.

The recondensation of Sn from the tube furnace on the nanoparticles is expected to be negligible considering the slight difference between the relative amount of Sn at room temperature and that at 1100 °C. Therefore, this method provides access to bimetallic Au/Sn compositions between 70/30 and 40/60 atomic %.

### Nanoparticle Phase Analysis

**Figure 3:**
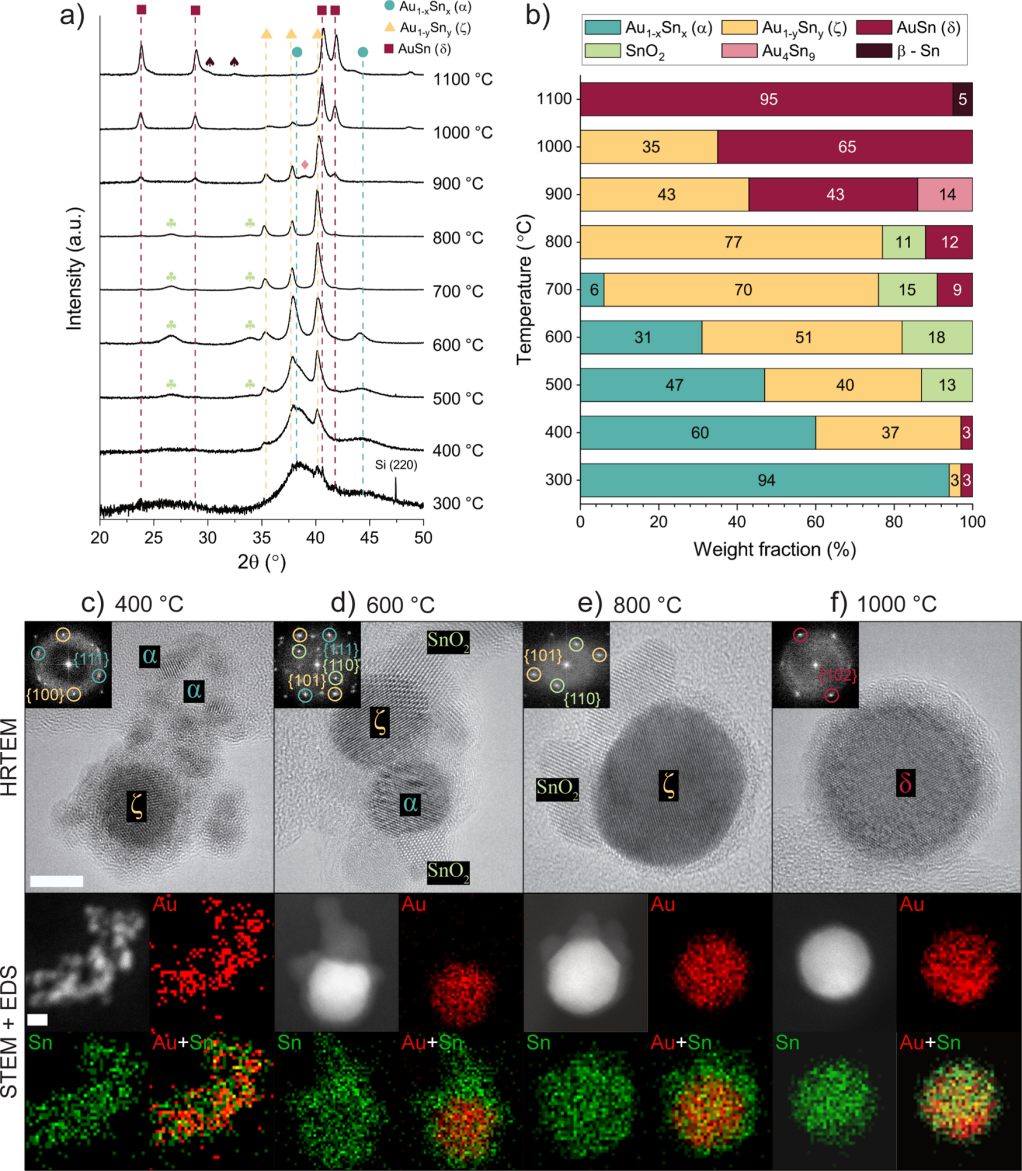
(a) PXRD measurements of Au–Sn nanoparticles as a function of the annealing temperature with the main peaks of each majority Au–Sn phase and minority phase (clovers, SnO_2_; diamonds, Au_4_Sn_9_; spades, β-Sn) indicated, and (b) weight fraction of each phase obtained from Rietveld refinements. HRTEM micrographs with FFTs in the insets and STEM micrographs and STEM-EDS elemental maps of 20 nm Au–Sn particles annealed at (c) 400, (d) 600, (e) 800, and (f) 1000 °C. Scale bars = 5 nm.

Further investigation of the effect of in-flight annealing on Au–Sn nanoparticles was conducted by analyzing their crystal structure as a function of the annealing temperature with PXRD (Figure 3a). Rietveld refinements of PXRD data provided the weight fraction of each phase observed (Figure 3b) (see the SI for structural details, Tables S2 and S3). Single-particle investigations were carried out on nanoparticles size selected to 20 nm using high-resolution (HR) TEM and scanning (S)TEM (Figure 3c–f).

A change of phase content in the Au–Sn nanoparticles is observed after in-flight annealing at different temperatures. Initially, the agglomerate particles consist, according to PXRD, up to 400 °C mainly of the Au-rich Au_1–*x*_Sn_*x*_ (*x* < 0.07) α-alloy.^45^ This phase is confirmed by the corresponding {111} planes observed in the fast Fourier transform (FFT) (Figure 3c). The small primary particle sizes of <5 nm, observed in HRTEM, explain the broadening of the peaks corresponding to the α-alloy phase in PXRD^46^ (see SI, Figure S2). In this temperature range and relative Sn composition of approximately 50 atomic % (Figure 2b), the δ-intermetallic phase^47^ should form, according to the bulk Au–Sn phase diagram.^36^ However, the α-alloy is obtained due to the short residence times of the annealing process, pointing toward a limited miscibility of Au and Sn in the initial synthesis stage.

Between 400 and 900 °C, the amount of Au_1–*y*_Sn_*y*_ (0.1 < *y* < 0.18) ζ-alloy phase^48^ increases toward it being the main phase from 600 to 800 °C. The corresponding {100} and {101} planes are detected in the FFTs alongside with an increase of particle size due to sintering (Figure 3c–e). Above 900 °C, larger amounts of the more Sn-rich AuSn δ-intermetallic phase are observed (Figure 3f), corresponding to {102} planes, and a reportedly metastable Au_4_Sn_9_ intermetallic phase^49^ is obtained. At 1100 °C, samples mainly consist of the δ-intermetallic phase in addition to small amounts of β-Sn.

Note that the relative Sn composition (Figure 2b) is inconsistent with the observed crystalline phases (Figure 3b) up to 700 °C. This observation can be explained by the high amount of amorphous Sn on the nanoparticles at low temperatures (FFT in Figure 3c), which could not be quantified with the available PXRD data. Indeed, as the temperature increases from 500 to 800 °C, a partial recrystallization of SnO_2_^50^ is observed on the surface of the nanoparticles (Figure 3b, 3d, and 3e), in accordance with reported formation conditions.^51−53^ From 800 °C, the relative Sn composition coincides with the phases observed in PXRD.

### Nanoparticle Size Effect

A qualitative discrepancy in the phase analysis by PXRD (Figure 3b) and HRTEM (Figure 3f) is observed for samples annealed at 1000 °C. Non-size-selected nanoparticles consist of 35 wt % ζ-alloy and 65 wt % δ-intermetallic phases based on PXRD, whereas 20 nm particles solely show the intermetallic phase in HRTEM. This difference can be explained by the effect of particle size on the spatial distribution of Au and Sn in the nanoparticles and thus on the corresponding phase formation. To study this nanoparticle size effect, 50 nm Au–Sn nanoparticles annealed at different temperatures were analyzed using TEM and compared to the corresponding 20 nm particles.

**Figure 4:**
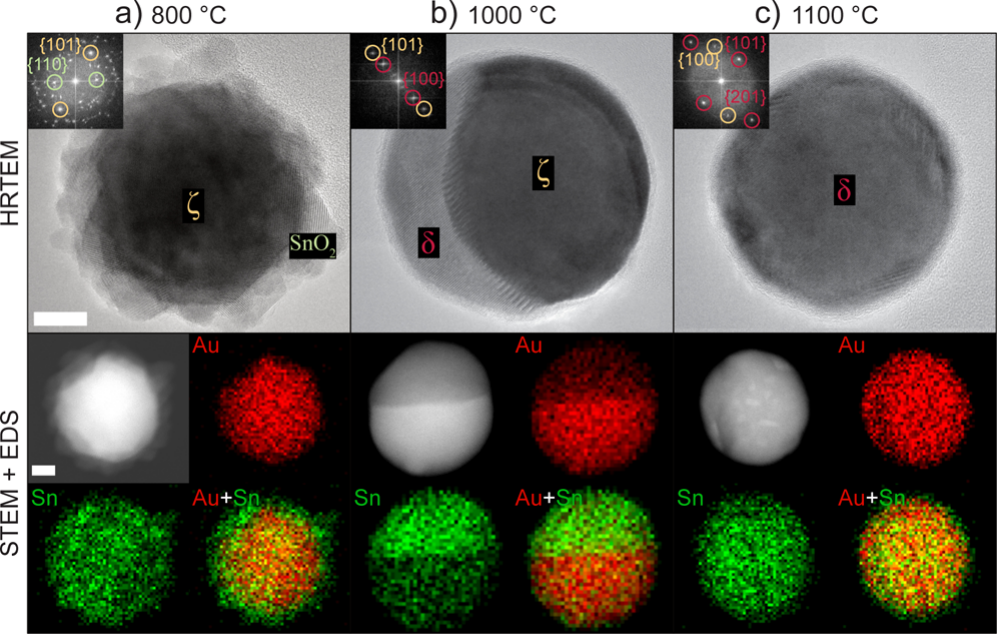
HRTEM micrographs with FFTs in the insets and STEM micrographs and STEM-EDS elemental maps of 50 nm Au–Sn particles annealed at (a) 800, (b) 1000, and (c) 1100 °C. Scale bars = 10 nm.

At 800 °C, 50 nm particles have a core–shell morphology with the ζ-alloy in the core and polycrystalline SnO_2_ in the shell (Figure 4a), similar to 20 nm particles (Figure 3e). The phase fractions of not size-selected Au–Sn nanoparticles (Figure 3b) correspond almost entirely to the ζ-alloy and SnO_2_, denoting a negligible size effect in this temperature range. At 1000 °C, however, 50 nm particles have a Janus morphology formed by the ζ-alloy and the δ-intermetallic phases (Figure 4b) while 20 nm particles only consist of the δ-intermetallic phase (Figure 3f). The occurrence of Janus-type particles is related to the inhomogeneous distribution of Sn oxides on the surface of the particles (see the following section).

A further increase in temperature to 1100 °C leads to 50 nm particles solely showing the δ-intermetallic phase (Figure 4c). Interestingly, a fainting signal of the ζ-alloy phase can still be observed in the FFT, and regions with different mass–thickness contrast can be noticed in STEM, where brighter areas correspond to more Au-rich locations. These observations suggest that a higher annealing temperature and/or a longer residence time are still necessary to complete the transformation to the δ-intermetallic phase.

Therefore, smaller 20 nm particles transform already at 1000 °C, whereas the transformation is completed at 1100 °C for 50 nm particles. This effect can be explained by the diffusion of Sn into the Au-rich ζ-alloy phase. Sn atoms need to diffuse over shorter distances for smaller particles. An increase in temperature is needed to enhance the diffusion of Sn and complete the transformation for larger particle sizes. Consequently, the nanoparticle morphology and phase fractions can be adjusted at high temperatures by controlling the nanoparticle size.

### Nanoparticle Surface Effect

**Figure 5:**
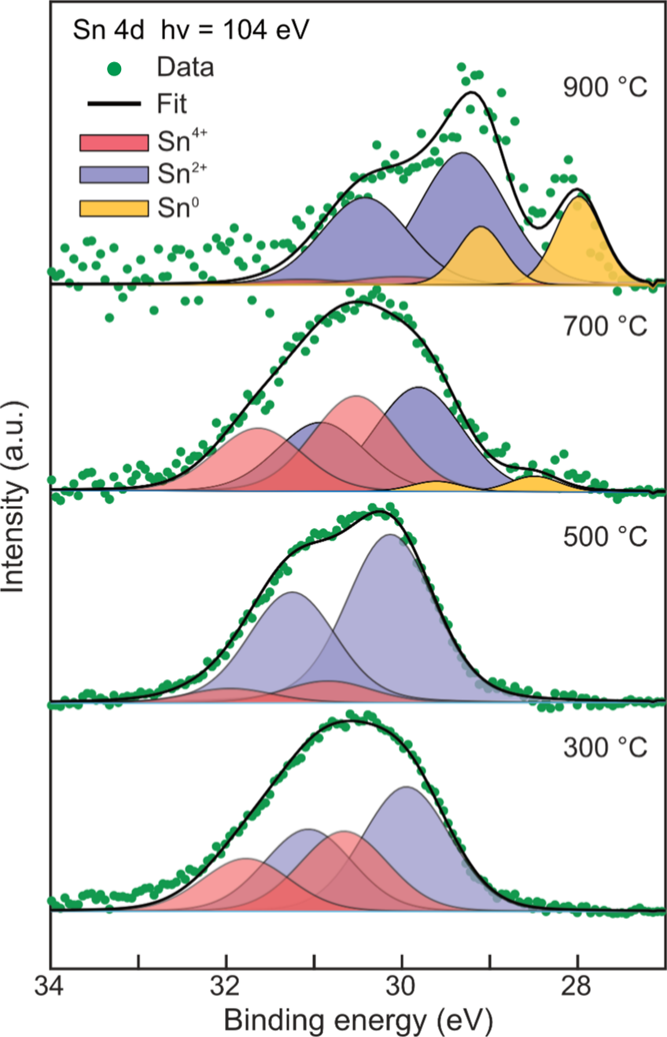
In-flight XPS measurements of Au–Sn nanoparticles measured at 300, 500, 700, and 900 °C (see the SI for peak fitting details).

The behavior of Sn on the surface of the nanoparticles is investigated to comprehend its enhanced diffusivity and thus the formation of more Sn-rich phases with the increase of annealing temperature. The chemical state of Sn (Sn 4d core level) on the surface of the nanoparticles was studied using in-flight XPS as a function of the annealing temperature (Figure 5).

Between 300 and 800 °C, Sn is found as Sn^4+^ (SnO_2_), consistent with PXRD (Figure 3b) and HRTEM (Figure 3d and 3e) results, and Sn^2+^ (SnO). These measurements are in line with previous works that reported a tendency of Sn oxides to be accumulated on the surface, regardless of the bulk composition and structure.^37,40^ Starting at 700 °C, an additional Sn^0^ signal is observed, pointing toward the reduction of Sn oxides to metallic Sn facilitated by the carrier gas atmosphere (N_2_ + 5 vol % H_2_).^54,55^ Additionally, around 700 °C, the evaporation of SnO, which has a higher vapor pressure than SnO_2_,^56^ seems to take place. This fact explains the decrease in relative Sn composition (Figure 2b) and the relative increase of Sn^4+^ signal at 700 °C. At 900 °C, the Sn^0^ signal increases alongside with the vanishing of Sn^4+^ signal, in agreement with the phase analysis results (Figure 3b), which no longer show reflections of SnO_2_.

Therefore, the increase in annealing temperature promotes the availability of metallic Sn and the formation of the δ-intermetallic phase. As Sn oxides are inhomogeneously distributed in the vicinity of the particles, e.g., annealed at 800 °C (Figure 3e and 4a), so is the δ-intermetallic phase formed at 1000 °C, resulting in particles with Janus morphology (Figure 4b). At higher annealing temperatures, the enhanced diffusivity of Sn causes the formation of 95 wt % δ-intermetallic phase (Figure 3b).

## Conclusions

In this work, a gas-phase synthesis method in combination with in-flight annealing and nanoparticle size selection has proven to be an effective way to tune Au–Sn nanoparticles. The method provided access to bimetallic Au/Sn ratios between 70/30 and 40/60 atomic % in the temperature range from 300 to 1100 °C. With the increase of in-flight annealing temperature, nanoparticles contained higher fractions of more Sn-rich phases from randomly ordered Au-rich alloys at lower temperatures to structurally fully ordered more Sn-rich intermetallics at higher temperatures. Sn oxides observed on the periphery of the nanoparticles have been found to reduce to metallic Sn starting at 700 °C, enabling its diffusion toward the Au-rich core. Additionally, above 900 °C, the nanoparticle size determined the morphology and phase fractions, where smaller nanoparticles have shown more Sn-rich phases at lower temperatures. This research provides insights in the in-flight annealing process of nanoparticles and the corresponding phase stability ranges. Thus, this method constitutes an effective approach to design nanoparticles with specific morphological, compositional, and structural properties.

## Supporting Information Available

Detailed experimental information about nanoparticle generation and processing, online and offline characterization, and detailed information about the results; log-normal fittings to the particle size distributions and the corresponding parameters for each temperature, a compilation of structural details of the known phases, as well as the results of the Rietveld refinements (PDF)
