# Supplementary material for: In-Flight Tuning of Au–Sn Nanoparticle Properties
Source: Langmuir. 2024 Jul 26;40(31):16393–9. doi: 10.1021/acs.langmuir.4c01656 (PMC11308768; doi:10.1021/acs.langmuir.4c01656)
Supplement: Supplementary file 2 — la4c01656_si_002.pdf [file la4c01656_si_002.pdf]

SUPPORTING INFORMATION

In-flight tuning of Au–Sn nanoparticle properties

Pau Ternero[†], Calle Preger[‡,¶], Axel Christian Eriksson[‡], Jenny Rissler[‡], Julia-Maria Hübner[*,§], and Maria E. Messing[*,†]

† Department of Physics and NanoLund, Lund University, 221 00 Lund, Sweden

‡ Department of Design Sciences and NanoLund, Lund University, 221 00 Lund, Sweden

¶ MAX IV Laboratory, Lund University, 221 00 Lund, Sweden

§ Faculty of Chemistry and Food Chemistry, TUD Dresden University of Technology, 01062 Dresden, Germany

E-mail: julia-maria.huebner@tu-dresden.de; maria.messing@ftf.lth.se

Number of pages: 8
Number of figures: 2
Number of schemes: 0
Number of tables: 3

1. Methods

1.1. Nanoparticle generation

1.2. Nanoparticle processing

1.3. Off-line characterization

1.4. On-line characterization

2. Results

2.1. Particle size distributions

2.2. Powder X-ray diffraction

1. Methods

1.1. Nanoparticle generation

A spark discharge generator (SDG) was employed to generate Au-Sn nanoparticles using spark ablation synthesis[1] (Figure 5a). In the SDG, Au and Sn rods (GoodFellow, ⌀ = 3.0 mm, > 99.95%) were placed face-to-face, with a gap of about 2 mm, serving as anode and cathode electrodes, respectively. The pressure in the system was kept at 1015 mbar (Bronkhorst, El-Press-Select). A 19 nF capacitor bank was continuously charged (10 kV, 10 mA), by means of a high-voltage power supply (Technix, Model CCR15- P-750), and discharged, producing a spark that repeatedly ablated the surface of the electrodes. The resulting supersaturated vapors consisting of electrode atoms rapidly cooled by adiabatic expansion, and the nucleation of atomic clusters began. The newborn particles grew by coalescence to form primary particles smaller than 5 nm. The coagulation and partial sintering of primary particles led to the formation of fractal-like agglomerates[2]. The generated Au-Sn nanoparticles were carried downstream using a gas mixture of N_2 + 5% H_2 (Linde, 99.9999%) with a flow rate of 1.68 L min^{-1} (Bronkhorst, El-Flow-Select).

1.2. Nanoparticle processing

A custom-built setup was used for the following annealing, size selection, and deposition of Au-Sn nanoparticles (Figure 5b). After production, the nanoparticles were conducted through a β-emitting 63Ni neutralizer, which guarantee a known charge distribution required for the later mobility diameter selection. After the neutralizer, a tube furnace (Lenton LTF, ceramic tube Alsint 99.7 type C 799, length of 60 cm, inner and outer diameter of 1.8 and 2.4 cm) was employed for the in-flight annealing study. When needed, the nanoparticles were size-selected, based on their electrical mobility diameter, by means of a differential mobility analyzer (DMA) (DMA2, custom Vienna type[3]), DMA1 was not used. Eventually, the nanoparticles were either counted by means of an electrometer or deposited by applying an electric field in a custom electrostatic precipitator (ESP). The pathway followed by the nanoparticles depended on the characterization technique that would be applied. Samples prepared for PXRD measurements required high particle concentrations to obtain decent signal-to-noise ratios. Therefore, the DMAs were bypassed, and the nanoparticles only went through the tube furnace before being deposited in the ESP. Samples produced for TEM analysis went through the tube furnace and DMA2, with which the desired nanoparticle size was selected.

1.3. On-line nanoparticle characterization

The electrometer (TSI 3086B) and the DMA2 were employed to record particle size distributions based on the electrical mobility diameter of the nanoparticles. A log-normal distribution is fitted to the data using a least squares approach[4] (Figure S1). The fitted parameters were the GMD, geometric standard deviation, and particle concentration of the distributions (Table S1).

In-flight XPS was performed at the gas-phase endstation of FinEstBeAMS beamline at MAX IV Laboratory using the aerosol sample delivery system (ASDS). The aerosol agglomerates entered the ASDS through a 100 μm critical orifice and formed a narrow and collimated beam through the

aerodynamic lens (PM1, Aerodyne). The narrow particle beam intersects the photon beam below the entrance of the electron analyzer. The XPS spectra was recorded using SCIENTA R4000 hemispherical electron analyzer rotated in vertical direction at incident photon energy of 104 eV with a vertical polarization. The photon energy was chosen to achieve the highest surface sensitivity (photoelectron kinetic energy ≅ 70 eV). The exit slit of the monochromator was set to 300 μm and the pass energy of the SCIENTA to 100 eV. Together with a 0.3 mm curved SCIENTA slit it provided an energy resolution for the experiments of approximately 75 meV. The binding energy scale is measured with respect to vacuum level and was calibrated using the outermost valence states of N_2 at 15.58 eV[5]. For the peak fitting, the Sn 4d core level is known to have a well-defined doublet separated by 0.7 eV. The Sn^0 peak is at 1.1 eV lower energy than Sn^{2+}, and the Sn^{4+} is shifted to 0.4 eV higher energy relative to Sn^{2+}[6,7]. The binding energy is measured with respect to vacuum level; hence the work function of the nanoparticle is included in the measured binding energy. Therefore, in the analysis, the position of the peaks was allowed to shift, as long as the relative shift between all peaks remained fixed.

A laser vaporizer AMS[8] was used to measure the effect of in-flight annealing on the nanoparticle Au/Sn ratio. AMS employs the same type of aerodynamic lens as the ASDS described above, followed by vaporization and electron impact (70 eV) ionization to enable time-of-flight mass spectrometry. The AMS was used in double vaporizer mode, where particles are vaporized by a 1064 nm intracavity laser (partial overlap with particle beam) combined with a tungsten surface heated to 600 °C (full overlap with particle beam). Both Au and Sn signals were confirmed to arise only from the laser vaporizer. Mass spectra were analyzed with SQUIRREL v1.66 and PIKA v.1.26. The collection efficiency, i.e. mass fraction of vaporized particles, and ionization efficiency, i.e., instrument sensitivity towards Au and Sn vapors, were both unconstrained[9]. Hence, absolute mass fractions were not available from the AMS dataset alone. To produce quantitative composition results from AMS, sensitivity to particle type and chemical species must be empirically determined. Ideally this is done in specific calibration experiments, but often it is done using literature values. It was apparent that Au sensitivity is lower than Sn sensitivity, which is likely due to a combination of lower ionization cross section and higher vaporization temperature for Au. Since we have neither calibration experiments nor literature values to rely on, we used the SEM-EDX results obtained at room temperature to rescale the AMS signal ratio to reflect atomic % composition. By applying a factor of 9.8 to the Au signal (keeping Sn signal as recorded) the SEM-EDX results at 20 °C were reproduced, allowing us to investigate the relative change of this ratio for different annealing temperatures (Figure 1a).

1.4. Off-line nanoparticle characterization

The nanoparticles were collected on a silicon wafer for SEM, on a Kapton foil for XRD, and on a TEM grid. The morphology and chemical composition of the Au-Sn nanoparticles was evaluated using SEM (Zeiss GeminiSEM 500) with an EDS detector (Oxford Instruments, Ultim Max, 170 mm^2). The crystal structure of the samples was studied with PXRD in transmission mode (Stoe Stadi MP, Mythen 1k detector, Cu K-α radiation, λ = 1.54178 Å). LaB_6 was measured as an external standard and used for refinement of instrument parameters, e.g., the zero shift. The diffractograms were then analyzed using the Jana2020 software[10]. The weight fractions of each phase obtained by Rietveld refinements for bulk samples were correlated with microscopic

observations of individual agglomerates/nanoparticles using TEM (Jeol JEM-3000F). For each sample, about 20 different agglomerates/nanoparticles were analyzed at distinct locations of the TEM grid. HRTEM was employed to determine the crystalline phases and their arrangement within the nanoparticles. STEM with a high-angle annular dark-field detector was utilized to obtain mass-thickness contrast information. Elemental data was obtained in STEM mode using an EDS detector (Oxford Instruments, X-Max, 80 mm^2). The micrographs were processed with ImageJ and the elemental information was treated with INCA and AZtec.

2. Results

2.1. Particle size distributions

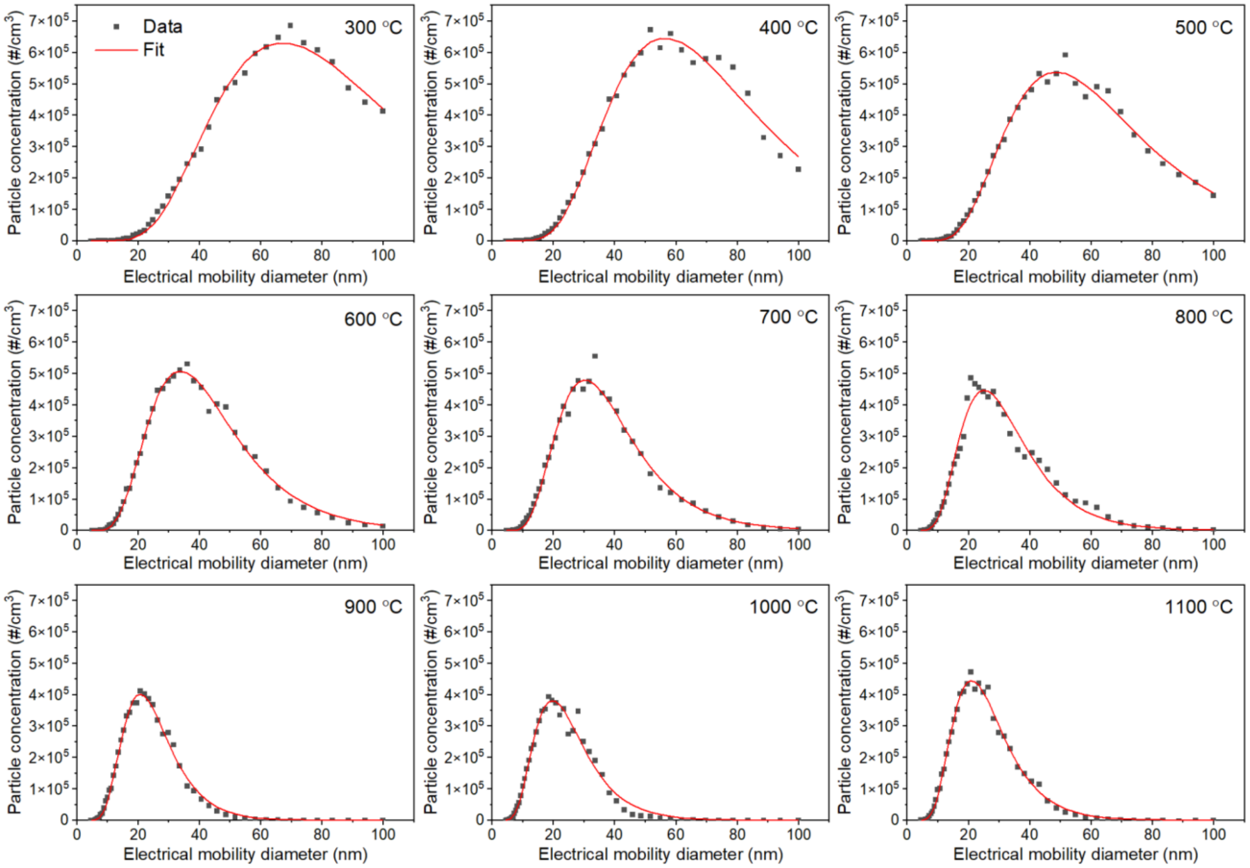

Figure S1. Particle number concentration as a function of the electrical mobility diameter for each annealing temperature. A log-normal distribution is fitted to the data using a least squares approach.

Table S1. GMD, geometric standard deviation, and particle concentration obtained from the log-normal distribution fitting to the particle size distributions as a function of the annealing temperature.

Temp. (°C)	300	400	500	600	700	800	900	1000	1100
GMD (nm)	67.06	56.82	48.87	33.92	30.53	24.71	20.83	19.89	21.05
σ_g	1.53	1.54	1.57	1.53	1.49	1.50	1.46	1.55	1.46
Conc. (#/cm^{-3})	29738 5.31	30670 4.52	26510 4.03	23390 4.23	21257 8.08	20367 2.05	16572 4.14	17899 8.97	18769 2.37

2.2. Powder X-ray diffraction

Table S2. Structural details for phases observed in the present study. Phase notations for the face centered cubic (*fcc*), hexagonal close packed (*hcp*) and nickeline phase are taken from the phase diagram[11].

**Phase**	**Composition**	**Structure Type**	**Space group**	**Pearsson symbol**
α[12]	$Au_{1-x}Sn_x$ (x<0.07)[11]	*fcc*-Cu/γ-Mn	*Fm*-3*m*	*cF*4
ζ[13]	$Au_{1-y}Sn_y$ (0.1<y<0.18)[11]	*hcp*-Mg	$P6_3/mmc$	*hP*2
δ[14]	AuSn	NiAs (nickeline)	$P6_3/mmc$	*hP*4
SnO_2[15]	SnO_2	TiO_2 (rutile)	$P4_2/mnm$	*tP*6
Au_4Sn_9[16]	Au_4Sn_9	γ-brass	*I*-43*m*	*cI*52
β-Sn[17]	Sn	β-Sn	$I4_1/amd$	*tI*4

Table S3. Rietveld refinement results including phase fractions, lattice parameters, and refinement details for investigated samples.

Sample	Annealing temp. (°C)	*α*-phase	*ζ*-phase	*δ*-phase	SnO_2	Au_4Sn_9	*β*-Sn	Refinement details
P32	300	>94%<br>*a*=4.034(1) Å	<3%	<3%				R_p=2.02, wR_p=2.63, *GOF*=1.25
P34	400	60%<br>a=4.0673(5) Å	37%<br>*a*=2.921(1) Å, *c*=4.757(2) Å	<3%				R_p=2.52, wR_p=3.34, *GOF*=1.67
P36	500	47%<br>*a*=4.0797(5) Å	40%<br>*a*=2.9311(3) Å, *c*=4.760(1) Å		13%<br>*a*=4.736(8) Å, *c*=3.21(1) Å			R_p=3.56, wR_p=4.87, *GOF*=2.69
P39	600	31%<br>*a*=4.095(2) Å	51%<br>*a*=2.922(1) Å, *c*=4.757(4) Å		18%<br>*a*=4.730(7) Å, *c*=3.197(6) Å			R_p=8.03, wR_p=11.45, *GOF*=6.61
P42	700	6%<br>*a*=4.115(1) Å	70%<br>*a*=2.9312(1) Å, *c*=4.7617(4) Å	9%<br>*a*=4.335(6) Å, *c*=5.471(5) Å	15%<br>*a*=4.741(9) Å, *c*=3.19(1) Å			R_p=5.73, wR_p=7.47, *GOF*=3.85
P45	800		77%<br>*a*=2.9388(1) Å, *c*=4.7581(2) Å	12%<br>*a*=4.311(1) Å, *c*=5.504(2) Å	11%<br>*a*=4.742(1) Å, *c*=3.194(2) Å			R_p=4.13, wR_p=5.53, *GOF*=2.76
P48	900		43%<br>*a*=2.9232(2) Å, *c*=4.7563(5) Å	43%<br>a=4.3228(6) Å, c=5.511(2) Å		14%<br>*a*=9.820(4) Å		R_p=3.05, wR_p=3.98, *GOF*=1.82
P52	1000		35%<br>*a*=2.9015(5) Å, *c*=4.742(1) Å	65%<br>*a*=4.3178(1) Å, *c*=5.529(3) Å				R_p=3.40, wR_p=4.39, *GOF*=1.98
P55	1100			95%<br>a=4.3069(2) Å, c=5.4950(4) Å			5%	R_p=4.36, wR_p=5.77, *GOF*=2.63

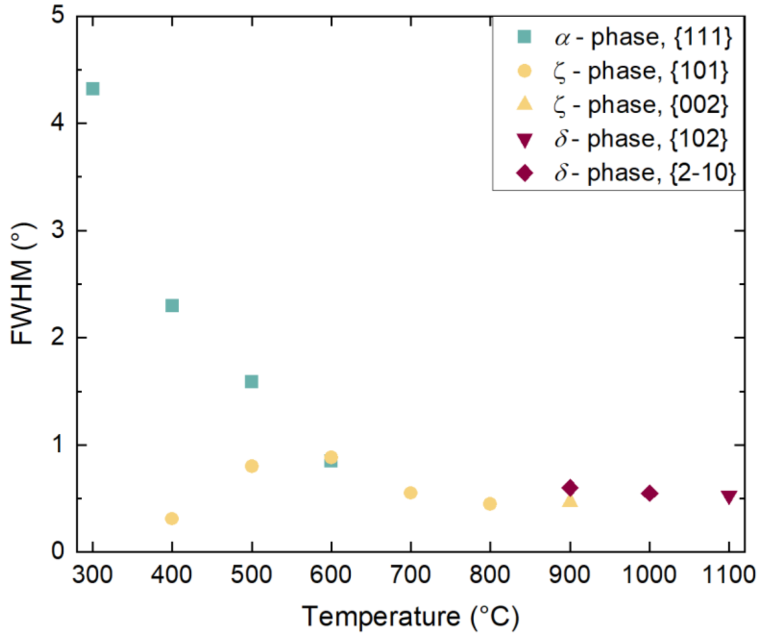

Figure S2. Full width at half maximum (FWHM) for specific reflections of observed phases versus annealing temperatures. Reflections with high intensity and without peak overlap were selected.

References

[1] Schmidt-Ott, A. *Spark Ablation: Building Blocks for Nanotechnology*; CRC Press, 2019.

[2] Feng, J.; Huang, L.; Ludvigsson, L.; Messing, M. E.; Maisser, A.; Biskos, G.; Schmidt-Ott, A. General approach to the evolution of singlet nanoparticles from a rapidly quenched point source. *J. Phys. Chem. C* **2016**, 120, 621–630.

[3] Knutson, E.; Whitby, K. Aerosol classification by electric mobility: apparatus, theory, and applications. *J. Aerosol Sci.* **1975**, 6, 443–451.

[4] Hinds, W. C.; Zhu, Y. *Aerosol technology: properties, behavior, and measurement of airborne particles*; John Wiley & Sons, 2022.

[5] Dutuit, O.; Carrasco, N.; Thissen, R.; Vuitton, V.; Alcaraz, C.; Pernot, P.; Balucani, N.; Casavecchia, P.; Canosa, A.; Le Picard, S.; others Critical review of N, N^+, N_2^+, N^{++}, and N_2^{++} main production processes and reactions of relevance to Titan's atmosphere. *Astrophys. J. Suppl. Ser.* **2013**, 204, 20.

[6] Themlin, J.; Sporken, R.; Darville, J.; Gaudano, R. JM Gilles, and RL Johnson. Resonant-photoemission study of SnO_2: cationic origin of the defect band-gap states. *Phys. Rev. B* **1990**, 42, 914.

[7] Akgul, F. A.; Gumus, C.; Ali, O. E.; Farha, A. H.; Akgul, G.; Ufuktepe, Y.; Liu, Z. Structural and electronic properties of SnO_2. *J. Alloys Compd.* **2013**, 579, 50–56.

[8] Nilsson, P. T.; Eriksson, A. C.; Ludvigsson, L.; Messing, M. E.; Nordin, E. Z.; Gudmundsson, A.; Meuller, B. O.; Deppert, K.; Fortner, E. C.; Onasch, T. B.; In-situ characterization of metal

nanoparticles and their organic coatings using laservaporization aerosol mass spectrometry. *Nano Res,* **2015**, 8, 3780–3795.
[9] Onasch, T.; Trimborn, A.; Fortner, E.; Jayne, J.; Kok, G.; Williams, L.; Davidovits, P.; Worsnop, D.; others Soot particle aerosol mass spectrometer: development, validation, and initial application. *Aerosol Sci. Technol.* **2012**, 46, 804–817.
[10] Petříček, V.; Palatinus, L.; Plášil, J.; Dušek, M. Jana2020 – a new version of the crystallographic computing system Jana. *Z. Kristallogr. – Cryst.* **2023**, 238, 271–282.
[11] Ciulik, J.; Notis, M. The Au-Sn phase diagram. *J. Alloys Compd*. **1993**,191, 71–78.
[12] Owen, E.; O'Donnell Roberts, E. The solubility of certain metals in gold. *J. Inst. Met.* **1945**, 71, 213–254.
[13] Massalski, T.; King, H. The lattice spacing relationships in close-packed α and ζ phases based on gold. *Acta Metall*. **1960**, 8, 677–683.
[14] Stenbeck, S.; Westgren, A. Roentgenanalyse der Gold-Zinn-Legierungen. *Z. Phys. Chem. B* **1931**, 14, 91–96.
[15] Baur, W. Ueber die Verfeinerung der Kristallstrukturbestimmung einiger Vertreter des Rutiltyps: TiO_2, SnO_2, GeO_2 und MgF_2. *Acta Crystallogr*. **1956**, 9, 515–520.
[16] Giessen, B. C. A metastable g-brass phase in the gold-tin system and a note on non-equilibrium hume-rothery phases. *Int. J. Mater. Res.* **1968**, 59, 805–809.
[17] Jette, E.; Foote, F. Precision determination of lattice constants. *J. Chem. Phys*. **1935**, 3, 605–616.
